# Nationwide trends in physical disability among rheumatoid arthritis patients in Korea (2010–2022): A retrospective cohort study based on national registry data

**DOI:** 10.1371/journal.pone.0362398

**Published:** 2026-11-04

**Authors:** Bo Young Kim, Ji-Won Kim, Hwajeong Lee, Jung-Yoon Choe, Sung-Hoon Park, Sang Gyu Kwak

**Affiliations:** 1 Division of Rheumatology, Department of Internal Medicine, School of Medicine, Daegu Catholic University, Daegu, Republic of Korea; 2 Department of Medical Statistics, School of Medicine, Daegu Catholic University, Daegu, Republic of Korea; Nippon Medical School, JAPAN

## Abstract

**Background:**

Whether the burden of legally certified disability among patients with rheumatoid arthritis (RA) has decreased in the modern treatment era is uncertain, particularly in Asia. We investigated trends in registered and physical disability among Korean patients with RA.

**Methods:**

Using National Health Insurance Service data (patient-level 50% random sample), we identified patients aged ≥19 years with seropositive RA registered in the copayment assistance program for rare and intractable diseases in 2010–2022. Disability status, including physical disability (certified musculoskeletal impairment), came from the national disability registry. We estimated annual prevalence and, in newly diagnosed patients, incidence per 1,000 person-years, and compared the results with national disability statistics.

**Results:**

The number of patients with seropositive RA identified in the database and included in this study was 33,992 in 2010 and 67,844 in 2022. The prevalence of registered disability declined from 20.3% to 14.6% and that of physical disability from 17.4% to 10.9% (both p for trend <0.001) in both sexes. Among 61,086 newly diagnosed patients, the incidence of registered disability did not change (5.08 per 1,000 person-years; annual change −0.9%, p = 0.24), whereas that of physical disability declined by 6.7% per year (p < 0.001). Men had a higher incidence of registered disability than women (6.73 vs. 4.58 per 1,000 person-years), but not of physical disability. Older age (odds ratio [OR] 1.035 per year, 95% CI 1.032–1.039), male sex (OR 1.295, 95% CI 1.174–1.429), and lower income were associated with incident registered disability. In 2022, physical disability remained more prevalent in RA (10.9%) than in the general population (2.3%) and in those aged ≥65 years (7.1%).

**Conclusions:**

Certified physical disability among Korean patients with RA declined substantially between 2010 and 2022, whereas the incidence of any registered disability remained stable. An excess burden of physical disability compared with the general population persisted.

## Introduction

Rheumatoid arthritis (RA) is a chronic inflammatory disease characterized by progressive joint destruction, functional limitation, and reduced quality of life, often leading to long-term disability [1]. Despite advances in pharmacologic therapy and earlier initiation of disease-modifying antirheumatic drugs (DMARDs), disability remains one of the most important long-term outcomes in RA and continues to contribute substantially to healthcare burden worldwide [2].

Functional disability in RA, usually measured with patient-reported instruments such as the Health Assessment Questionnaire, reflects the cumulative effects of persistent inflammation, structural joint damage, and comorbid conditions, and remains a central outcome in both clinical practice and population-based research [3]. Early disease characteristics and the timing of effective treatment strongly influence long-term functional outcomes [4,5].

Over the past two decades, the management of RA has undergone major transformation with the widespread adoption of treat-to-target strategies and the introduction of biologic and targeted synthetic DMARDs [6]. These advances have significantly improved disease control and reduced radiographic progression in many patients. Several studies conducted in Western countries have reported declining rates of functional and work-related disability in the modern treatment era [7,8], whereas others have found little change in permanent work disability [9]. Disability remains a persistent concern, particularly among older patients, women, and those with long-standing disease.

Global epidemiologic data indicate that the prevalence of RA has steadily increased over recent decades, largely due to aging populations and improved survival [10,11]. As individuals live longer with RA, the cumulative burden of disability becomes increasingly important to evaluate.

Although temporal trends in RA-related disability have been examined in several Western countries, differences in healthcare systems, treatment accessibility, and demographic characteristics may influence long-term outcomes across regions [9]. Therefore, findings from Western populations may not be directly generalizable to Asian populations. In Korea, the nationwide health insurance database can be linked to the national disability registry, in which disability is certified by physicians against statutory criteria [12]. Registered disability differs from the functional disability measured by questionnaires: it reflects established, persistent impairment that meets legally defined thresholds, and physical disability is the registry category that captures musculoskeletal impairment. This provides a valuable opportunity to evaluate long-term trends in certified disability within a universal healthcare system.

Another important consideration is the comparison of disability burden between patients with RA and the general population. While improvements in RA management may reduce disability rates, it remains unclear whether these improvements parallel broader societal trends or whether RA-related disability continues to exceed that observed in the general population.

Therefore, the objective of this nationwide population-based study was to investigate temporal trends in registered disability, and specifically physical disability, among patients with RA in Korea from 2010 to 2022. Specifically, we evaluated trends in the prevalence of disability in the prevalent RA population and in the incidence of disability among newly diagnosed patients, sex-specific patterns, factors associated with the development of disability, and differences in disability burden compared with the general population.

## Materials and methods

### Data source and study population

We conducted a nationwide, population-based study using data from the Korean National Health Insurance Service (KNHIS), which covers approximately 97.7% of the South Korean population (around 50 million individuals); the remainder are covered by the Medical Aid program, which is also captured in the database. The KNHIS database includes comprehensive information on demographics, diagnoses, procedures, and prescriptions [13]. We identified adults aged ≥19 years with seropositive RA between Jan 1, 2010, and Dec 31, 2022. RA cases were defined using both the International Classification of Diseases, 10th Revision (ICD-10) diagnostic code for seropositive RA (M05) and registration in the copayment assistance program for rare and intractable diseases (V code). Registration in this program requires confirmation of diagnosis by a physician according to standardized criteria, thereby enhancing diagnostic validity [14]; therefore, all patients in the study population met both criteria.

We restricted the primary analysis to seropositive RA because the combination of the seropositive RA code and program registration provides the most specific case definition available in Korean claims data, and because seropositive disease is associated with a more erosive course and is therefore most relevant to certified structural disability; a previous nationwide Korean study of disability in RA likewise focused on seropositive RA [15]. Patients with seronegative or unspecified RA (ICD-10 M06) registered in the same program were identified in the same manner and analyzed separately as a sensitivity analysis. Unless otherwise specified, the study population and “patients with RA” hereafter refer to patients with seropositive RA.

A full extraction of all eligible patients over the 13-year study period exceeded the maximum data volume that can be extracted from the KNHIS research database for a single project; therefore, a 50% random sample, the maximum extraction permitted, was used. Sampling was performed at the patient level: a sampled patient remained in the study population in every calendar year in which the patient received RA-related care with a valid program registration. The same individual could therefore contribute to more than one calendar year, and each annual population was treated as a separate cross-section in the prevalence analyses.

Two analytic populations were defined. The prevalent RA population in each calendar year comprised all sampled patients receiving RA-related care in that year and was used to estimate annual prevalence. The incident RA cohort comprised the 61,086 sampled patients newly diagnosed with RA during the study period (new patients; Table 1); these patients were followed from the year of diagnosis until 2022 and were used to estimate the incidence of disability.

**Table 1:** Characteristics of patients with seropositive rheumatoid arthritis and prevalence of registered disability and physical disability, 2010–2022.

Year	Patients with RA, n	Women, n (%)	Age, years, mean (SD)	Newly diagnosed patients, n	Age at diagnosis, years, mean (SD)	Registered disability, n (%)	Physical disability, n (%)
2010	33,992	27,950 (82.2)	56.8 (12.7)	4,863	54.2 (13.3)	6,901 (20.3)	5,903 (17.4)
2011	37,177	30,525 (82.1)	57.2 (12.7)	4,702	54.7 (13.5)	7,253 (19.5)	6,156 (16.6)
2012	40,319	33,044 (82.0)	57.6 (12.7)	4,596	55.5 (13.6)	7,576 (18.8)	6,390 (15.8)
2013	43,260	35,309 (81.6)	58.1 (12.7)	4,559	55.7 (13.7)	7,841 (18.1)	6,567 (15.2)
2014	46,162	37,582 (81.4)	58.6 (12.7)	4,593	56.2 (13.5)	8,231 (17.8)	6,837 (14.8)
2015	48,664	39,448 (81.1)	59.1 (12.7)	4,589	56.4 (13.9)	8,390 (17.2)	6,903 (14.2)
2016	51,220	41,446 (80.9)	59.5 (12.7)	4,710	56.9 (13.7)	8,573 (16.7)	7,030 (13.7)
2017	53,890	43,553 (80.8)	60.0 (12.7)	4,520	57.0 (13.8)	8,838 (16.4)	7,125 (13.2)
2018	56,669	45,726 (80.7)	60.5 (12.7)	4,755	57.4 (14.0)	9,069 (16.0)	7,188 (12.7)
2019	59,843	48,214 (80.6)	60.9 (12.7)	4,809	57.7 (13.7)	9,427 (15.8)	7,356 (12.3)
2020	62,170	49,899 (80.3)	61.4 (12.7)	4,656	57.7 (13.8)	9,583 (15.4)	7,347 (11.8)
2021	65,192	52,217 (80.1)	61.8 (12.7)	4,939	58.3 (14.2)	9,815 (15.1)	7,412 (11.4)
2022	67,844	54,267 (80.0)	62.2 (12.7)	4,795	58.0 (13.9)	9,899 (14.6)	7,406 (10.9)
p for trend						<0.001	<0.001

RA, rheumatoid arthritis; SD, standard deviation. Patients with RA denotes the prevalent RA population in each calendar year (patient-level 50% random sample). Newly diagnosed patients are the patients diagnosed with RA in each year, who form the incident RA cohort (total n = 61,086). Percentages of registered disability and physical disability use the prevalent RA population of the same year as the denominator. p for trend, Cochran–Armitage test.

### Korean national disability registration system

Disability status was obtained from the national disability registry, which was established under the Act on Welfare of Persons with Disabilities and is administered by the Ministry of Health and Welfare [12]. Registration is voluntary and is initiated by the individual or a guardian, who applies at a local community service center with a disability diagnosis issued by a qualified specialist physician together with supporting investigations. The documents are reviewed by the National Pension Service against statutory criteria before registration and severity are determined. Disability is assessed only after the impairment is judged to be permanent, generally after at least six months of adequate treatment, and reassessment may be required. Fifteen disability categories are recognized: physical, brain lesion, visual, hearing, speech, facial, kidney, cardiac, liver, respiratory, ostomy, epilepsy, intellectual, autistic, and mental disability [12].

Physical disability (*jiche* disability) is the category covering musculoskeletal impairment and is certified on the basis of amputation, joint impairment (including ankylosis or restricted range of motion of the joints of the upper and lower limbs), limb dysfunction, or spinal impairment and deformity. In patients with RA, physical disability therefore represents certified, persistent impairment of the joints or limbs. In this study, registered disability (any disability) refers to a valid registration in any of the 15 categories, and physical disability refers to registration in the physical category; for patients with more than one registered impairment, the main disability type recorded in the registry was used. Severity was graded on a six-level scale until June 2019 and has been classified as severe or not severe since July 2019 [12]; all analyses used registration irrespective of severity.

Registration provides access to disability-related benefits that depend on severity and household income, including a disability pension or disability allowance, rehabilitation and personal-assistance services, support for medical expenses, tax deductions, and discounts on public services and transportation [12]. Registered disability is therefore a legally certified, administratively defined outcome and is distinct from the functional disability measured with patient-reported instruments.

### Outcomes and variables

The primary outcomes were registered disability and physical disability. Prevalent disability in a given year was defined as a valid registration in that year among patients in the prevalent RA population. Incident disability was defined as a first disability registration after RA diagnosis among patients in the incident RA cohort. Time to disability was calculated from the date of RA diagnosis to the date of first disability registration. Covariates included age, sex, and income level, which was measured by the insurance-contribution level in 21 categories (0, Medical Aid beneficiaries; 1–20, ascending vigintiles of the insurance premium).

### General population reference data

For comparison, national counts of registered persons with disabilities by age, sex, and disability category were obtained from the annual disability registration statistics of the Ministry of Health and Welfare [16], and the year-end resident registration population was used as the denominator. Because registered disability increases steeply with age and patients with RA were older than the general population, we also calculated prevalence in the general population aged ≥65 years [17]. This group is older than the RA population in every study year and therefore provides a conservative reference that tends to underestimate rather than overestimate any excess associated with RA.

### Statistical analysis

Descriptive statistics are presented as numbers and proportions for categorical variables and means with standard deviations (SDs) for continuous variables. Temporal trends in prevalence across all 13 annual values were assessed using the Cochran–Armitage test for trend, overall and separately in men and women. Trends in mean age were assessed using linear regression of the annual means on calendar year, weighted by the inverse of their variance. Sex differences in prevalence were compared using the chi-square test and expressed as prevalence ratios.

Incidence was expressed per 1,000 person-years in the incident RA cohort. In each calendar year, person-time was calculated as the number of cohort members under observation and free of disability registration at the start of the year, plus half of those newly diagnosed and minus half of those newly registered during that year. Trends in incidence were assessed using Poisson regression with the logarithm of person-time as an offset and are expressed as annual percent changes; incidence was compared between sexes using incidence rate ratios (IRRs) with the exact Poisson test. Because the first study year contained only the first partial year of follow-up of the first entry cohort and yielded an unstable estimate, incidence trends were estimated for 2011–2022. To examine whether the incidence trend changed during the study period, we fitted segmented (joinpoint) Poisson regression models with a single joinpoint at each candidate year between 2013 and 2020 and compared the best-fitting model with a model without a joinpoint using a parametric bootstrap likelihood-ratio test (5,000 replicates).

Factors associated with incident disability were examined using multivariable logistic regression in the incident RA cohort, with first disability registration during follow-up as the dependent variable and age (continuous), sex, and income level (continuous) as covariates. Results are reported as odds ratios (ORs) with 95% confidence intervals (CIs). Individual-level analyses were performed using SAS version 9.4 (SAS Institute, Cary, NC, USA) and Stata version 18.0 (Stata Corp, College Station, TX, USA) within the KNHIS analysis environment, and analyses of the aggregated outputs and national statistics were performed using R version 4.6.1 (R Foundation for Statistical Computing, Vienna, Austria). A two-sided p value of less than 0.05 was considered statistically significant.

Several sensitivity analyses were performed (S3 Table). First, prevalence was standardized to the sex distribution of the 2010 RA population. Age-specific counts were not available in the aggregated outputs, so formal age standardization was not possible, and the comparison with the general population aged ≥65 years was used instead to account for age. Second, the prevalence and incidence analyses were repeated in the seronegative or unspecified RA (M06) population and in the combined M05 and M06 population, to assess the influence of shifts in diagnostic coding. Third, because dates of death were not available and person-time could not be censored at death, the incidence trend analysis was repeated after reducing person-time under explicit mortality assumptions, using the all-cause mortality reported for Korean patients with incident seropositive RA (12.8 per 1,000 person-years) [15] distributed across ages with a Gompertz function, and at two and three times that rate. Fourth, incidence trends were re-estimated using quasi-Poisson models to allow for overdispersion. Finally, to illustrate the effect of the denominator, the annual number of newly registered disabilities was also expressed as a proportion of the prevalent RA population in the same year.

### Ethics statement

This study was approved by the Institutional Review Board of Daegu Catholic University Hospital (DCUMC 2025-01-027) and conducted in accordance with the Declaration of Helsinki and its amendments. The requirement for informed consent was waived because the KNHIS database provides fully anonymized and de-identified data.

## Results

### Study population

The number of patients with seropositive RA identified in the database and included in this study increased from 33,992 in 2010–67,844 in 2022, whereas the annual number of newly diagnosed patients remained stable at approximately 4,500–4,900 (Table 1). The annual net increase in the prevalent population (2,327–3,185 patients) was therefore smaller than the number of new patients, implying that approximately 1,450–2,350 patients left the prevalent population each year. Over the same period, the number of patients with seronegative or unspecified RA (M06) decreased from 62,373–39,631, so the combined M05 and M06 population increased by only 11.5% (from 96,365–107,475), and the proportion coded as seropositive increased from 35.3% to 63.1% (S3 Table). The mean age of the prevalent RA population increased from 56.8 (SD 12.7) to 62.2 (12.7) years and the mean age at RA diagnosis from 54.2 (13.3) to 58.0 (13.9) years, whereas the proportion of women decreased slightly from 82.2% to 80.0% (Table 1).

### Trends in disability prevalence

Among patients with seropositive RA, the prevalence of registered disability declined from 20.3% in 2010 to 14.6% in 2022 and that of physical disability from 17.4% to 10.9% (both p for trend <0.001) (Table 1, Fig 1). Prevalence standardized to the 2010 sex distribution was virtually identical to crude prevalence (14.5% and 10.9% in 2022; S3 Table).

**Fig 1:**
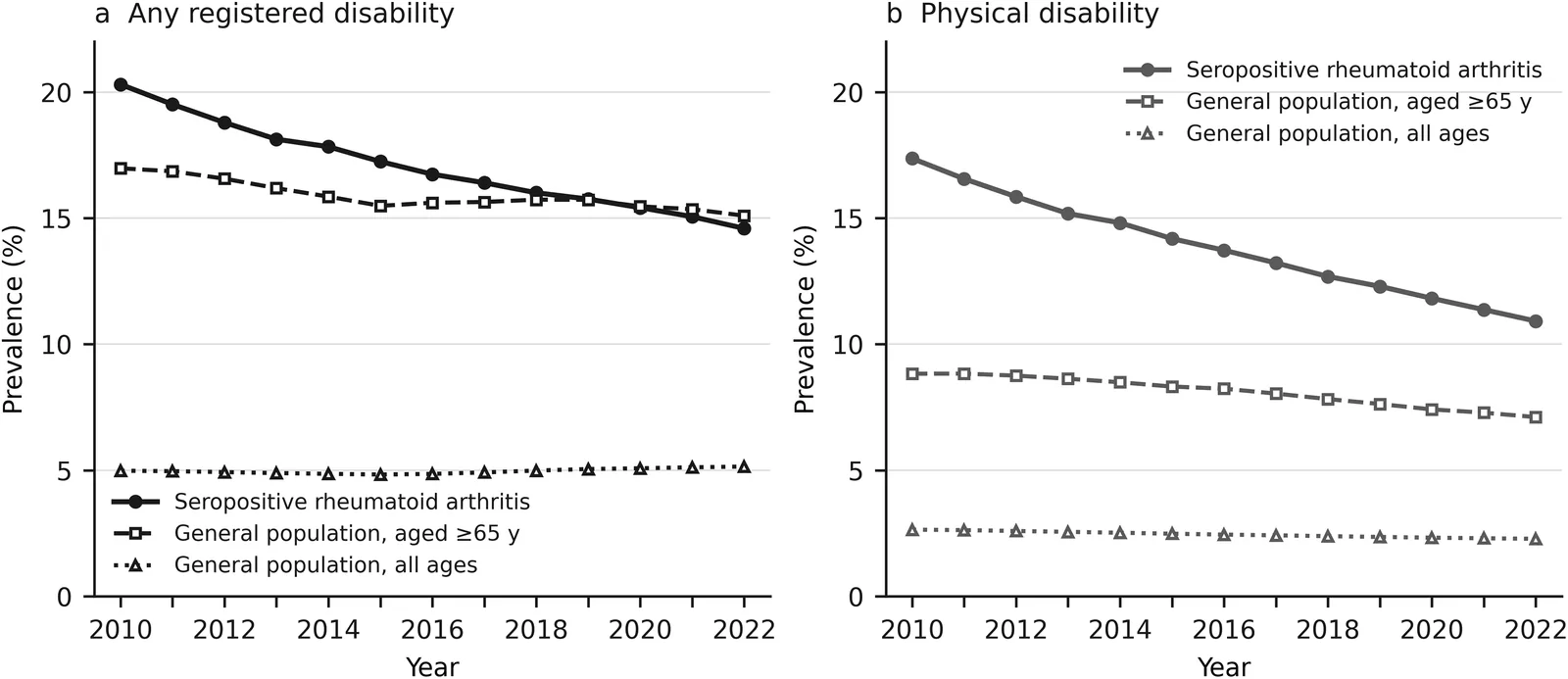
Prevalence of registered disability among patients with seropositive rheumatoid arthritis and in the Korean general population, 2010–2022. (a) Any registered disability. (b) Physical disability. Solid lines with filled circles, patients with seropositive rheumatoid arthritis; dashed lines with open squares, general population aged ≥65 years; dotted lines with open triangles, general population of all ages. General population values were calculated from the annual disability registration statistics of the Ministry of Health and Welfare, with the year-end resident registration population as the denominator.

### Sex-specific prevalence

Among patients with seropositive RA, the prevalence of registered disability declined in both men (from 21.7% to 16.3%) and women (from 20.0% to 14.2%), as did that of physical disability (men, from 16.6% to 10.4%; women, from 17.5% to 11.1%) (all p for trend <0.001; Table 4, S1 Table). In 2022, registered disability was more prevalent in men than in women (16.3% [2,212/13,577] vs. 14.2% [7,687/54,267]; prevalence ratio 1.15, p < 0.001), whereas physical disability was slightly more prevalent in women (11.1% vs. 10.4%; prevalence ratio for men 0.94, p = 0.022). Among patients with a registered disability, physical disability accounted for a greater proportion in women than in men (78.0% vs. 63.6%, p < 0.001).

### Incidence of disability among newly diagnosed patients

Among the 61,086 patients newly diagnosed with seropositive RA, 1,948 first disability registrations occurred over 383,346 person-years between 2011 and 2022, an incidence of 5.08 per 1,000 person-years (Table 2, Fig 2). The incidence of registered disability did not change significantly over time (annual change −0.9%, 95% CI −2.3% to 0.6%; p = 0.24). In contrast, the incidence of physical disability (947 events over 387,451 person-years; 2.44 per 1,000 person-years) declined from 4.47 per 1,000 person-years in 2011 to 1.75 in 2022, an annual decline of 6.7% (95% CI 4.8% to 8.6%; p < 0.001). The decline was observed in both men (9.3% per year) and women (5.9% per year) (both p < 0.001). Joinpoint analysis identified no significant change in the trend of either outcome (p = 0.11 for registered disability and p = 0.48 for physical disability; S3 Table).

**Table 2:** Incidence of registered disability and physical disability among patients with newly diagnosed seropositive rheumatoid arthritis, 2010–2022.

Year	Incident cohort at risk, cumulative n	Any disability, events	Person-years	Rate per 1,000 PY	Physical disability, events	Person-years	Rate per 1,000 PY	Any disability, men, per 1,000 PY	Any disability, women, per 1,000 PY	Physical disability, men, per 1,000 PY	Physical disability, women, per 1,000 PY	New registrations / prevalent RA, %
2010^a^	4,863	57	2,403	23.72	37	2,413	15.33	51.44	16.69	30.55	11.45	0.17
2011	9,565	41	7,136	5.75	32	7,161	4.47	6.76	5.48	4.69	4.41	0.11
2012	14,161	57	11,736	4.86	39	11,774	3.31	8.38	3.90	5.15	2.81	0.14
2013	18,720	82	16,244	5.05	59	16,303	3.62	6.47	4.65	3.91	3.54	0.19
2014	23,313	114	20,722	5.50	77	20,811	3.70	6.27	5.28	3.65	3.72	0.25
2015	27,902	118	25,198	4.68	68	25,330	2.68	5.07	4.57	3.47	2.45	0.24
2016	32,612	136	29,720	4.58	87	29,902	2.91	4.97	4.46	3.48	2.74	0.27
2017	37,132	205	34,164	6.00	78	34,434	2.27	9.05	5.07	2.61	2.16	0.38
2018	41,887	204	38,598	5.29	86	38,990	2.21	6.97	4.77	2.40	2.15	0.36
2019	46,696	241	43,157	5.58	104	43,676	2.38	7.16	5.10	2.22	2.43	0.40
2020	51,352	220	47,659	4.62	88	48,313	1.82	6.32	4.08	1.98	1.77	0.35
2021	56,291	284	52,204	5.44	128	53,002	2.41	7.99	4.63	2.63	2.35	0.44
2022	61,086	246	56,806	4.33	101	57,755	1.75	5.62	3.91	1.34	1.88	0.36
2011–2022, pooled		1,948	383,346	5.08	947	387,451	2.44	6.73	4.58	2.60	2.40	
Annual change, % (95% CI)				−0.9 (−2.3 to 0.6)			−6.7 (−8.6 to −4.8)	+0.1 (−2.6 to 2.8)	−1.5 (−3.2 to 0.3)	−9.3 (−12.9 to −5.6)	−5.9 (−8.1 to −3.7)	
p for trend				0.24			<0.001	0.97	0.10	<0.001	<0.001	

PY, person-years; CI, confidence interval. Person-time in each year was the number of cohort members under observation and free of disability registration at the start of the year, plus half of those newly diagnosed and minus half of those newly registered during the year. Annual change and p for trend were estimated by Poisson regression over 2011–2022. ^a^The year 2010 contained only the first partial year of follow-up of the first entry cohort and was excluded from the trend and pooled estimates. The last column shows the annual number of new registrations of any disability divided by the prevalent RA population of the same year, shown for comparison; because the numerator is drawn from the accumulating incident cohort whereas the denominator includes patients diagnosed before 2010, it rises over time without any change in risk.

**Fig 2:**
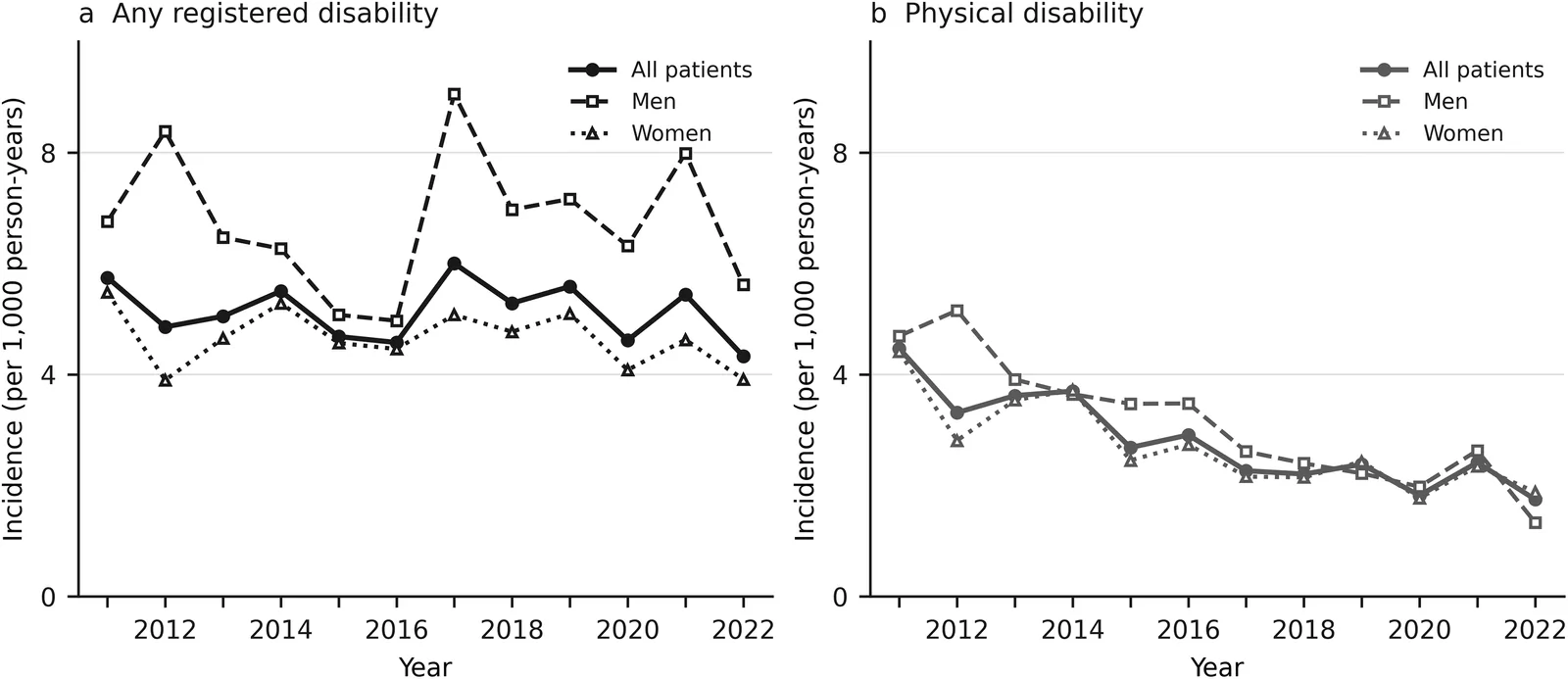
Incidence of registered disability among 61,086 patients with newly diagnosed seropositive rheumatoid arthritis, 2011–2022. (a) Any registered disability. (b) Physical disability. Rates are per 1,000 person-years, with person-time accrued from the year of diagnosis. The year 2010 is not shown because it contained only the first partial year of follow-up of the first entry cohort. Solid lines with filled circles, all patients; dashed lines with open squares, men; dotted lines with open triangles, women.

Men had a higher incidence of registered disability than women (6.73 vs. 4.58 per 1,000 person-years; IRR 1.47, 95% CI 1.34–1.62; p < 0.001), whereas the incidence of physical disability did not differ significantly by sex (2.60 vs. 2.40 per 1,000 person-years; IRR 1.08, 95% CI 0.93–1.25; p = 0.28).

When the annual number of newly registered disabilities was instead divided by the prevalent RA population of the same year, the resulting proportion rose from 0.17% in 2010 to 0.44% in 2021 (Table 2), with an apparent change point in 2017 (p < 0.001; S3 Table). This pattern arose because the numerator was drawn from the incident cohort, whose at-risk population accumulated from 4,863 patients in 2010–61,086 in 2022, whereas the denominator also included patients diagnosed before 2010; it does not reflect an increase in the risk of disability.

### Comparison with the general population

In the general population, the prevalence of registered disability changed little (4.98% in 2010 and 5.15% in 2022), whereas that of physical disability decreased from 2.65% to 2.28% (Table 3, Fig 1). Disability was more prevalent among patients with seropositive RA than in the general population throughout the study period: in 2022, registered disability was 2.8 times and physical disability 4.8 times more prevalent in RA (4.1 and 6.6 times, respectively, in 2010).

**Table 3:** Prevalence of registered disability and physical disability among patients with seropositive rheumatoid arthritis and in the Korean general population, 2010–2022.

Year	RA: registered disability, %	General population: registered disability, %	General population ≥65 years: registered disability, %	RA: physical disability, %	General population: physical disability, %	General population ≥65 years: physical disability, %
2010	20.3	4.98	17.0	17.4	2.65	8.8
2011	19.5	4.97	16.9	16.6	2.63	8.8
2012	18.8	4.93	16.6	15.8	2.60	8.8
2013	18.1	4.89	16.2	15.2	2.56	8.6
2014	17.8	4.86	15.9	14.8	2.52	8.5
2015	17.2	4.83	15.5	14.2	2.49	8.3
2016	16.7	4.86	15.6	13.7	2.45	8.2
2017	16.4	4.92	15.6	13.2	2.42	8.0
2018	16.0	4.99	15.7	12.7	2.39	7.8
2019	15.8	5.05	15.7	12.3	2.36	7.6
2020	15.4	5.08	15.5	11.8	2.33	7.4
2021	15.1	5.12	15.4	11.4	2.31	7.3
2022	14.6	5.15	15.1	10.9	2.28	7.1
Ratio, RA / reference, 2010		4.1	1.20		6.6	1.97
Ratio, RA / reference, 2022		2.8	0.97		4.8	1.54

RA, rheumatoid arthritis. General population values were calculated from the annual disability registration statistics of the Ministry of Health and Welfare, with the year-end resident registration population as the denominator; for the population aged ≥65 years, the denominator was the resident population multiplied by the proportion aged ≥65 years. All trends, p for trend <0.001 (Cochran–Armitage test).

Because patients with seropositive RA were older than the general population, we also compared them with the general population aged ≥65 years. Compared with this older reference, the prevalence of registered disability in RA was similar in 2022 (14.6% vs. 15.1%; ratio 0.97; 1.20 in 2010), whereas physical disability remained 1.54 times more prevalent (10.9% vs. 7.1%; 1.97 in 2010). The relative decline in physical disability between 2010 and 2022 was greater in RA (37%) than in the general population (14%) or in the general population aged ≥65 years (19%).

### Distribution of disability types

In 2022, physical disability accounted for 74.8% of registered disabilities among patients with seropositive RA, followed by hearing (10.8%), visual (6.2%), brain lesion (2.8%), and kidney (1.7%) disability (S4 Table). In the national disability registration statistics for the same year, physical disability accounted for 44.4% of all registered disabilities and for 47.1% among registered persons aged ≥65 years [16].

### Age of patients with disability

The mean age of patients with registered disability increased from 62.4 (SD 10.7) to 68.6 (10.6) years in men and from 61.5 (11.1) to 69.0 (10.9) years in women (Table 4, S2 Table), corresponding to 0.55 and 0.63 years per calendar year, respectively (both p < 0.001); these increases were greater than that of the entire RA population (0.46 years per year). Similar increases were observed among patients with physical disability (0.49 and 0.60 years per year in men and women, respectively; both p < 0.001).

**Table 4:** Sex-specific prevalence of disability and age of patients with disability among patients with seropositive rheumatoid arthritis, 2010 and 2022.

	2010, men	2010, women	2022, men	2022, women
Patients with RA, n	6,042	27,950	13,577	54,267
Registered disability, n (%)	1,311 (21.7)	5,590 (20.0)	2,212 (16.3)	7,687 (14.2)
Physical disability, n (%)	1,005 (16.6)	4,898 (17.5)	1,407 (10.4)	5,999 (11.1)
Physical disability among patients with registered disability, %	76.7	87.6	63.6	78.0
Age of patients with registered disability, mean (SD)	62.4 (10.7)	61.5 (11.1)	68.6 (10.6)	69.0 (10.9)
Age of patients with physical disability, mean (SD)	61.7 (10.5)	61.3 (10.9)	67.5 (9.9)	68.6 (10.4)
Incident registered disability, n	25	32	78	168
Age at first registration, mean (SD)	57.5 (14.6)	62.2 (11.1)	65.8 (9.3)	62.9 (13.0)
Incident physical disability, n	15	22	19	82
Age at first registration, mean (SD)	53.6 (12.2)	60.9 (11.1)	62.9 (9.3)	57.6 (12.1)

RA, rheumatoid arthritis; SD, standard deviation. Ages of patients with registered or physical disability are the mean ages, in each year, of all patients living with that registration (prevalent cases). Age at first registration refers to incident cases in the incident RA cohort. Annual values for all years are shown in S1 and S2 Tables.

Among incident cases, the mean age at first registration of any disability increased in men (from 57.5 to 65.8 years; 0.49 years per year, p = 0.014) and slightly in women (from 62.2 to 62.9 years; 0.21 years per year, p = 0.033). The numbers of incident cases of physical disability in each sex and year were small (Table 4).

### Time to physical disability

Among the 984 patients in the incident cohort who developed physical disability, the mean time from RA diagnosis to registration was 4.36 years (SD 3.27). This interval was slightly shorter in men than in women (4.03 [3.07] vs. 4.48 [3.33] years; p = 0.053). Because follow-up was limited to a maximum of 13 years, these intervals describe disability that developed within the study period.

### Factors associated with incident disability

In multivariable logistic regression in the incident cohort (n = 59,826 with complete data; 1,941 events), older age (OR 1.035 per year, 95% CI 1.032–1.039; p < 0.001), male sex (OR 1.295, 95% CI 1.174–1.429; p < 0.001), and lower income level (OR 0.991 per one-category increase, 95% CI 0.984–0.998; p = 0.010) were independently associated with incident registered disability (Table 5). Income level was not significantly associated with incident disability in univariable analysis (p = 0.24).

**Table 5:** Multivariable logistic regression analysis of factors associated with incident registered disability in the incident seropositive rheumatoid arthritis cohort.

Variable	OR	95% CI	p value
Age, per year	1.035	1.032–1.039	<0.001
Sex, men vs. women	1.295	1.174–1.429	<0.001
Income level, per one-category increase (0–20)	0.991	0.984–0.998	0.010

OR, odds ratio; CI, confidence interval. Dependent variable: first registration of any disability during follow-up. n = 59,826 patients with complete data (1,941 events); 1,260 patients were excluded because of missing values. Income level: 0, Medical Aid beneficiaries; 1–20, ascending vigintiles of the insurance premium. C statistic, 0.643.

## Discussion

Concerns about long-term disability are among the most common questions raised by patients with RA during clinical care, underscoring the importance of understanding population-level disability trends [18]. In this nationwide population-based study spanning more than a decade, the prevalence of registered disability and physical disability among patients with RA in Korea declined substantially from 2010 to 2022. Among newly diagnosed patients, the incidence of physical disability declined by approximately 7% per year, whereas the incidence of any registered disability remained stable. Despite these improvements, disability remained more prevalent among patients with RA than in the general population, and an excess of physical disability persisted even in comparison with the general population aged ≥65 years.

The decline in disability observed in this study is consistent with findings from Western countries that have reported reductions in functional and work-related disability in the era of biologic therapies and treat-to-target strategies [7,8], although not all studies have shown a decline in work disability [9,19]. In our study, the relative decline in physical disability was greater than that in the general population, and the excess of physical disability over the general population persisted throughout the study period, emphasizing the continuing burden of musculoskeletal impairment associated with RA. Registered disability differs from the work disability and questionnaire-based functional disability examined in most previous studies; it reflects certified, persistent structural or functional impairment and is less influenced by labor market conditions, which may partly explain differences between studies.

Several factors are likely to have contributed to the decline in prevalence. First, the prevalent RA population expanded substantially. The annual number of newly diagnosed patients was stable, but the number of prevalent patients doubled, which suggests that the expansion reflected the accumulation of patients who maintained program registration and shifts in diagnostic coding rather than a true doubling of RA in Korea; consistent with this, the number of patients with seronegative or unspecified RA decreased over the same period, and the combined population increased by only 11.5%. Patients who entered the prevalent population after 2010 had a much lower burden of disability than those already present—only approximately 3% of newly diagnosed patients acquired a disability registration during follow-up—so the expansion of the denominator diluted the prevalence of disability. Second, patients with long-standing disability, who were older, may have left the prevalent population because of death or discontinuation of RA-related care. Dates of death were not available, so we could not distinguish between these possibilities; recovery from pre-existing disability is unlikely to have been a major factor because registration is granted only for impairment judged to be permanent. Third, the incidence of physical disability declined among newly diagnosed patients, indicating a genuine reduction in the risk of developing certified musculoskeletal impairment. Importantly, prevalence also declined in the combined seropositive and seronegative population (S3 Table), indicating that the decline was not explained by coding shifts alone.

When the annual number of newly registered disabilities is expressed as a proportion of the prevalent RA population, it appears to increase and then plateau. This pattern is an artifact of the denominator, because new registrations arose only from patients diagnosed during the study period, whose number at risk increased every year. When incidence was expressed per person-year in the incident cohort, the incidence of any registered disability was stable and that of physical disability declined, without a significant change point. The stable incidence of any registered disability despite the decline in physical disability reflects an increasing contribution of non-physical categories, such as hearing, visual, and kidney disability, which are more closely related to aging and comorbidity than to RA itself.

The decline in physical disability coincided with the wider adoption of treat-to-target strategies and advanced therapies. However, the present study did not include information on disease activity, the timing of diagnosis and treatment, or DMARD exposure, so the observed trends cannot be attributed to specific changes in treatment. Whether earlier diagnosis and timely treatment escalation reduce the risk of certified disability at the population level should be evaluated in future studies incorporating clinical and treatment data.

The mean age of patients with registered disability increased over time, more rapidly than that of the RA population as a whole. This shift may reflect delayed development of disability, but it may also reflect aging of the RA population, later diagnosis, and the survival of patients with long-standing disability; our data cannot distinguish between these explanations. Delayed disability development has also been reported in longitudinal cohort studies in the biologic era [8]. Age remained the strongest determinant of incident disability in our multivariable analysis.

The sex-specific findings require careful interpretation. Because RA predominantly affects women, women accounted for most patients with disability, and physical disability accounted for a larger proportion of registered disabilities in women than in men. Per patient, however, men had a higher prevalence and incidence of registered disability, and male sex was independently associated with incident registered disability. The excess in men was largely attributable to non-physical categories, because the incidence of physical disability did not differ significantly between the sexes. A male predominance is also observed in the national disability registry, in which men accounted for 57.9% of registered persons with disabilities in 2022 [16], and may reflect a higher burden of comorbidities such as cardiovascular disease, kidney disease, and hearing impairment in men. These findings do not contradict previous reports that women with RA have more severe disease manifestations, worse functional outcomes, and lower remission rates [20–23], because questionnaire-based functional limitation alone is not sufficient to meet the criteria for registered disability.

Lower income level was independently associated with incident registered disability, although the effect was small (approximately 17% lower odds across the full range of income levels). Because registration provides access to income-dependent benefits, this association may reflect a greater incentive to register among patients with lower income, as well as differences in access to care; the present data cannot distinguish between these explanations.

According to the national disability registration statistics, men accounted for 57.9% of registered persons with disabilities in Korea in 2022, and physical disability was the most common category (44.4%), followed by hearing (16.0%), visual (9.5%), brain lesion (9.2%), and intellectual (8.5%) disability [16]. These national figures are not derived from the present study population. Among patients with RA in the present study, physical disability accounted for a substantially larger proportion of registered disabilities (74.8%), followed by hearing (10.8%), visual (6.2%), brain lesion (2.8%), and kidney (1.7%) disability (S4 Table). Although the proportion of physical disability declined in both groups over the study period, it was consistently 30–32 percentage points higher in RA than in the general population in every year from 2010 (85.5% vs. 53.1%) to 2022 (74.8% vs. 44.4%) (S4 Table). This difference is consistent with the predominantly musculoskeletal impairment associated with RA.

Direct comparisons between the disability profile of patients with RA and that of the general Korean population should be interpreted with caution for several reasons. First, the study population comprised patients with RA, whereas the national disability statistics represent the entire Korean population, resulting in substantial differences in age and sex distributions; we addressed the age difference in part by comparison with the population aged ≥65 years. Second, disability in the general population arises from a wide range of congenital, traumatic, neurological, and chronic medical conditions, whereas disability in this study reflects outcomes occurring within a population already affected by RA. Third, registration as a person with disability is influenced by administrative criteria and healthcare access, which may differ between disease-specific cohorts and the general population. Finally, because the present analysis is based on claims and disability registration data rather than direct functional assessments, the observed disability patterns may not fully reflect the true burden of functional impairment in patients with RA.

Several limitations of this study should be considered when interpreting the findings. First, the KNHIS database provides anonymized and de-identified administrative data, and therefore individual diagnoses could not be directly verified at the patient level. However, the use of the copayment assistance program for rare and intractable diseases (V code), which requires confirmation of diagnosis by a physician according to standardized criteria, likely enhanced diagnostic accuracy and minimized potential misclassification. This structured registration system has been widely used in nationwide epidemiologic studies in Korea and is considered a reliable approach for identifying patients with RA.

Second, we were unable to determine the specific causes of physical disability recorded in the national disability registry. Physical disability may arise from various causes unrelated to RA, including traumatic injuries, osteoarthritis, surgical complications, or congenital conditions. Nevertheless, the predominance of physical disability among patients with RA and its excess over an older general population suggest that RA is a major contributor to the observed disability burden, although this cannot be established for individual patients.

Third, detailed data on disease activity, radiographic damage, and treatment, including the type and timing of DMARDs, were not analyzed. Consequently, we could not evaluate the association between treatment patterns and trends in disability outcomes. Future studies incorporating longitudinal clinical and medication data will be important to clarify the relationship between treatment and long-term functional outcomes.

Fourth, dates of death were not available, so person-time could not be censored at death, which biases incidence rates downward. In sensitivity analyses under explicit mortality assumptions, the decline in the incidence of physical disability persisted (6.1% per year in the base case and 4.9% per year at three times the assumed mortality; both p < 0.001), and attrition accounted for approximately 9% (at most 27%) of the observed decline (S3 Table). Fifth, age-specific counts were not available, so formal age standardization of the comparison with the general population was not possible; we therefore used the general population aged ≥65 years as a conservative reference. Sixth, registration is voluntary and is influenced by administrative criteria and incentives, and the severity grading system changed in 2019; although our definition did not depend on severity, changes in the propensity to register cannot be excluded. Seventh, the logistic regression model did not account for differences in follow-up time and was available only for registered disability overall, not for physical disability specifically. Finally, the date of diagnosis was derived from claims and program registration and may not correspond to the onset of disease.

This study has several important strengths. The use of a nationwide healthcare database enabled evaluation of long-term disability trends in a large and representative population covering nearly the entire Korean population. The extended study period allowed assessment of temporal changes over more than a decade, providing valuable insight into evolving patterns of disability in the contemporary treatment era. In addition, the incidence analysis based on person-time in newly diagnosed patients, together with national disability statistics for the entire population and for older adults, provided meaningful context for interpreting the relative burden of disease.

In conclusion, the prevalence of registered disability and physical disability among patients with RA in Korea declined substantially between 2010 and 2022, and the incidence of physical disability declined among newly diagnosed patients, whereas the incidence of any registered disability remained stable. Physical disability remained more common than in the general population, even in comparison with older adults. Older age, male sex, and lower income level were associated with incident registered disability. Continued monitoring of disability using appropriate person-time denominators, and future research incorporating detailed clinical information, including disease activity, medication exposure, and cause-specific disability data, will be essential to clarify the mechanisms underlying these trends and to identify patients at the highest risk.

## Supporting information

S1 Table Annual sex-specific prevalence of registered disability and physical disability among patients with seropositive rheumatoid arthritis, 2010–2022. (DOCX)

S2 Table Annual mean age of patients with registered disability and physical disability among patients with seropositive rheumatoid arthritis, by sex, 2010–2022. (DOCX)

S3 Table Sensitivity analyses. (A) Sex-standardized prevalence. (B) Seronegative or unspecified RA (M06) and combined M05 and M06 populations. (C) Incidence trends under mortality assumptions. (D) Joinpoint and quasi-Poisson analyses.
(DOCX)

S4 Table Distribution of registered disability types among patients with seropositive rheumatoid arthritis and in the Korean general population, 2010–2022. (A) Patients with seropositive rheumatoid arthritis. (B) General population, all ages. (C) General population aged ≥65 years.
(DOCX)

S1 Dataset Aggregated data underlying the tables and figures. (XLSX)
