# Supplementary material for: Nationwide trends in physical disability among rheumatoid arthritis patients in Korea (2010–2022): A retrospective cohort study based on national registry data
Source: PLoS One. 2026 Nov 4;21(11):e0362398. doi: 10.1371/journal.pone.0362398 (PMC13734594; doi:10.1371/journal.pone.0362398)
Supplement: Supplementary file 1 [file pone.0362398.s001.docx]

**S1 Table. Annual sex-specific prevalence of registered disability and physical disability among patients with seropositive rheumatoid arthritis, 2010–2022.**

| **Year** | **Men, n** | **Men: registered disability, n (%)** | **Men: physical disability, n (%)** | **Men: physical among registered, %** | **Women, n** | **Women: registered disability, n (%)** | **Women: physical disability, n (%)** | **Women: physical among registered, %** |
| --- | --- | --- | --- | --- | --- | --- | --- | --- |
| 2010 | 6,042 | 1,311 (21.7) | 1,005 (16.6) | 76.7 | 27,950 | 5,590 (20.0) | 4,898 (17.5) | 87.6 |
| 2011 | 6,652 | 1,378 (20.7) | 1,050 (15.8) | 76.2 | 30,525 | 5,875 (19.2) | 5,106 (16.7) | 86.9 |
| 2012 | 7,275 | 1,422 (19.5) | 1,069 (14.7) | 75.2 | 33,044 | 6,154 (18.6) | 5,321 (16.1) | 86.5 |
| 2013 | 7,951 | 1,508 (19.0) | 1,123 (14.1) | 74.5 | 35,309 | 6,333 (17.9) | 5,444 (15.4) | 86.0 |
| 2014 | 8,580 | 1,639 (19.1) | 1,210 (14.1) | 73.8 | 37,582 | 6,592 (17.5) | 5,627 (15.0) | 85.4 |
| 2015 | 9,216 | 1,680 (18.2) | 1,218 (13.2) | 72.5 | 39,448 | 6,710 (17.0) | 5,685 (14.4) | 84.7 |
| 2016 | 9,774 | 1,749 (17.9) | 1,248 (12.8) | 71.4 | 41,446 | 6,824 (16.5) | 5,782 (14.0) | 84.7 |
| 2017 | 10,337 | 1,847 (17.9) | 1,298 (12.6) | 70.3 | 43,553 | 6,991 (16.1) | 5,827 (13.4) | 83.4 |
| 2018 | 10,943 | 1,921 (17.6) | 1,317 (12.0) | 68.6 | 45,726 | 7,148 (15.6) | 5,871 (12.8) | 82.1 |
| 2019 | 11,629 | 2,038 (17.5) | 1,377 (11.8) | 67.6 | 48,214 | 7,389 (15.3) | 5,979 (12.4) | 80.9 |
| 2020 | 12,271 | 2,113 (17.2) | 1,400 (11.4) | 66.3 | 49,899 | 7,470 (15.0) | 5,947 (11.9) | 79.6 |
| 2021 | 12,975 | 2,192 (16.9) | 1,424 (11.0) | 65.0 | 52,217 | 7,623 (14.6) | 5,988 (11.5) | 78.6 |
| 2022 | 13,577 | 2,212 (16.3) | 1,407 (10.4) | 63.6 | 54,267 | 7,687 (14.2) | 5,999 (11.1) | 78.0 |
| p for trend |  | <0.001 | <0.001 |  |  | <0.001 | <0.001 |  |
| Prevalence ratio, men vs. women, 2010 |  | 1.08 (p=0.003) | 0.95 (p=0.10) |  |  |  |  |  |
| Prevalence ratio, men vs. women, 2022 |  | 1.15 (p<0.001) | 0.94 (p=0.022) |  |  |  |  |  |

p for trend, Cochran–Armitage test; prevalence ratios were compared with the chi-square test.
