# Supplementary material for: Nationwide trends in physical disability among rheumatoid arthritis patients in Korea (2010–2022): A retrospective cohort study based on national registry data
Source: PLoS One. 2026 Nov 4;21(11):e0362398. doi: 10.1371/journal.pone.0362398 (PMC13734594; doi:10.1371/journal.pone.0362398)
Supplement: Supplementary file 2 [file pone.0362398.s002.docx]

**S2 Table. Annual mean age of patients with registered disability and physical disability among patients with seropositive rheumatoid arthritis, by sex, 2010–2022.**

| **Year** | **Registered disability, men** | **Registered disability, women** | **Physical disability, men** | **Physical disability, women** | **Incident disability, men: n; age** | **Incident disability, women: n; age** | **Incident physical disability, men: n; age** | **Incident physical disability, women: n; age** |
| --- | --- | --- | --- | --- | --- | --- | --- | --- |
| 2010 | 62.4 (10.7) | 61.5 (11.1) | 61.7 (10.5) | 61.3 (10.9) | 25; 57.5 (14.6) | 32; 62.2 (11.1) | 15; 53.6 (12.2) | 22; 60.9 (11.1) |
| 2011 | 62.7 (10.9) | 62.0 (11.1) | 62.0 (10.7) | 61.9 (10.9) | 10; 67.5 (9.4) | 31; 58.2 (11.8) | 7; 65.9 (10.2) | 25; 59.4 (11.3) |
| 2012 | 63.1 (10.9) | 62.8 (11.0) | 62.6 (10.6) | 62.6 (10.8) | 21; 62.2 (14.5) | 36; 58.1 (10.1) | 13; 58.5 (15.9) | 26; 60.0 (10.0) |
| 2013 | 63.6 (10.8) | 63.5 (11.0) | 63.2 (10.5) | 63.3 (10.8) | 23; 54.8 (12.9) | 59; 62.3 (15.1) | 14; 58.1 (11.4) | 45; 60.0 (14.2) |
| 2014 | 64.0 (10.8) | 64.3 (11.0) | 63.7 (10.5) | 64.1 (10.8) | 29; 60.9 (12.5) | 85; 61.6 (12.1) | 17; 60.8 (13.7) | 60; 61.0 (11.1) |
| 2015 | 64.5 (10.8) | 64.8 (10.9) | 64.1 (10.5) | 64.7 (10.7) | 29; 60.8 (12.8) | 89; 61.8 (12.6) | 20; 57.4 (11.1) | 48; 59.6 (11.8) |
| 2016 | 65.0 (10.7) | 65.3 (10.9) | 64.4 (10.2) | 65.1 (10.6) | 34; 63.5 (10.1) | 102; 62.0 (11.8) | 24; 59.5 (13.4) | 63; 60.5 (11.7) |
| 2017 | 65.8 (10.7) | 66.0 (10.9) | 65.1 (10.2) | 65.7 (10.6) | 72; 64.9 (12.4) | 133; 62.4 (12.9) | 21; 59.3 (12.9) | 57; 56.5 (11.6) |
| 2018 | 66.7 (10.6) | 66.7 (10.9) | 65.9 (10.1) | 66.4 (10.5) | 63; 64.5 (9.0) | 141; 63.7 (13.4) | 22; 59.9 (7.2) | 64; 57.7 (11.7) |
| 2019 | 67.1 (10.6) | 67.2 (11.0) | 66.2 (10.1) | 66.8 (10.6) | 73; 63.3 (11.9) | 168; 62.3 (15.0) | 23; 60.6 (11.5) | 81; 57.2 (14.6) |
| 2020 | 67.6 (10.5) | 67.9 (10.9) | 66.6 (10.0) | 67.4 (10.6) | 72; 65.4 (11.6) | 148; 62.4 (12.4) | 23; 60.3 (11.5) | 65; 57.5 (12.1) |
| 2021 | 68.1 (10.5) | 68.5 (11.0) | 66.9 (10.1) | 68.1 (10.5) | 101; 65.0 (9.9) | 183; 62.3 (12.5) | 34; 61.1 (9.6) | 94; 57.7 (11.9) |
| 2022 | 68.6 (10.6) | 69.0 (10.9) | 67.5 (9.9) | 68.6 (10.4) | 78; 65.8 (9.3) | 168; 62.9 (13.0) | 19; 62.9 (9.3) | 82; 57.6 (12.1) |
| Change, years per calendar year (p) | 0.55 (<0.001) | 0.63 (<0.001) | 0.49 (<0.001) | 0.60 (<0.001) | 0.49 (0.014) | 0.21 (0.033) | — | — |

Values are mean (SD) age in years. Registered and physical disability columns refer to all patients living with the registration in each year (prevalent cases); incident columns refer to the age at first registration in the incident RA cohort. Trends were not estimated for incident physical disability because the annual numbers by sex were small. Change per calendar year was estimated by inverse-variance weighted linear regression. For comparison, the mean age of the entire prevalent RA population increased by 0.46 years per calendar year (p<0.001).
