# Supplementary material for: Nationwide trends in physical disability among rheumatoid arthritis patients in Korea (2010–2022): A retrospective cohort study based on national registry data
Source: PLoS One. 2026 Nov 4;21(11):e0362398. doi: 10.1371/journal.pone.0362398 (PMC13734594; doi:10.1371/journal.pone.0362398)
Supplement: Supplementary file 3 [file pone.0362398.s003.docx]

**S3 Table. Sensitivity analyses.** (A) Sex-standardized prevalence. (B) Seronegative or unspecified RA (M06) and combined M05 and M06 populations. (C) Incidence trends under mortality assumptions. (D) Joinpoint and quasi-Poisson analyses.

**A. Prevalence standardized to the sex distribution of the 2010 RA population**

| **Year** | **Women, %** | **Registered disability, crude %** | **Registered disability, sex-standardized %** | **Physical disability, crude %** | **Physical disability, sex-standardized %** |
| --- | --- | --- | --- | --- | --- |
| 2010 | 82.2 | 20.30 | 20.30 | 17.37 | 17.37 |
| 2011 | 82.1 | 19.51 | 19.51 | 16.56 | 16.56 |
| 2012 | 82.0 | 18.79 | 18.79 | 15.85 | 15.85 |
| 2013 | 81.6 | 18.13 | 18.12 | 15.18 | 15.19 |
| 2014 | 81.4 | 17.83 | 17.82 | 14.81 | 14.82 |
| 2015 | 81.1 | 17.24 | 17.23 | 14.19 | 14.20 |
| 2016 | 80.9 | 16.74 | 16.72 | 13.73 | 13.74 |
| 2017 | 80.8 | 16.40 | 16.37 | 13.22 | 13.23 |
| 2018 | 80.7 | 16.00 | 15.97 | 12.68 | 12.70 |
| 2019 | 80.6 | 15.75 | 15.72 | 12.29 | 12.30 |
| 2020 | 80.3 | 15.41 | 15.37 | 11.82 | 11.83 |
| 2021 | 80.1 | 15.06 | 15.01 | 11.37 | 11.38 |
| 2022 | 80.0 | 14.59 | 14.54 | 10.92 | 10.93 |

Age-specific counts were not available in the aggregated outputs, so age standardization could not be performed; the comparison with the general population aged ≥65 years (Table 3) was used instead.

**B. Seronegative or unspecified RA (M06) and combined M05 and M06 populations**

| **Year** | **M05, n** | **M06, n** | **Combined, n** | **M05 share, %** | **M06: registered disability, %** | **M06: physical disability, %** | **Combined: registered disability, %** | **Combined: physical disability, %** |
| --- | --- | --- | --- | --- | --- | --- | --- | --- |
| 2010 | 33,992 | 62,373 | 96,365 | 35.3 | 15.4 | 11.4 | 17.1 | 13.5 |
| 2011 | 37,177 | 62,036 | 99,213 | 37.5 | 15.0 | 11.1 | 16.7 | 13.2 |
| 2012 | 40,319 | 59,853 | 100,172 | 40.2 | 14.3 | 10.5 | 16.1 | 12.7 |
| 2013 | 43,260 | 57,144 | 100,404 | 43.1 | 14.1 | 10.4 | 15.8 | 12.5 |
| 2014 | 46,162 | 54,747 | 100,909 | 45.7 | 14.0 | 10.2 | 15.8 | 12.3 |
| 2015 | 48,664 | 52,394 | 101,058 | 48.2 | 13.7 | 10.0 | 15.4 | 12.0 |
| 2016 | 51,220 | 48,715 | 99,935 | 51.3 | 13.5 | 9.7 | 15.2 | 11.8 |
| 2017 | 53,890 | 45,315 | 99,205 | 54.3 | 13.3 | 9.5 | 15.0 | 11.5 |
| 2018 | 56,669 | 43,868 | 100,537 | 56.4 | 13.3 | 9.1 | 14.8 | 11.1 |
| 2019 | 59,843 | 42,820 | 102,663 | 58.3 | 13.2 | 8.8 | 14.7 | 10.8 |
| 2020 | 62,170 | 40,062 | 102,232 | 60.8 | 12.8 | 8.5 | 14.4 | 10.5 |
| 2021 | 65,192 | 40,641 | 105,833 | 61.6 | 12.6 | 8.4 | 14.1 | 10.2 |
| 2022 | 67,844 | 39,631 | 107,475 | 63.1 | 12.6 | 8.2 | 13.9 | 9.9 |
| p for trend |  |  |  |  | <0.001 | <0.001 | <0.001 | <0.001 |

The M06 population was identified and sampled in the same manner as the M05 population. Incidence in the M06 incident cohort, 2011–2022: registered disability 4.46 per 1,000 person-years, annual change +1.3% (95% CI 0.2 to 2.4; p=0.022); physical disability 1.61 per 1,000 person-years, annual change −5.0% (95% CI −6.7 to −3.3; p<0.001). In the M06 multivariable model, the OR for men was 1.445 (95% CI 1.343–1.555) and for income level 0.991 (p<0.001).

**C. Incidence trends (2011–2022) under mortality assumptions**

| **Outcome** | **Mortality assumption** | **Rate per 1,000 PY** | **Person-time lost in 2022, %** | **Annual change, % (95% CI)** | **p value** |
| --- | --- | --- | --- | --- | --- |
| any registered disability | No mortality (main analysis) | 5.08 | 0.0 | -0.9 (-2.3 to 0.6) | 0.24 |
| any registered disability | Constant 12.8 per 1000 PY | 5.37 | 7.6 | -0.3 (-1.7 to 1.2) | 0.73 |
| any registered disability | Age-dependent, mean 12.8 per 1000 PY | 5.33 | 7.3 | -0.2 (-1.7 to 1.3) | 0.78 |
| any registered disability | Age-dependent, mean 25.7 per 1000 PY (2x) | 5.58 | 13.9 | +0.4 (-1.0 to 1.9) | 0.56 |
| any registered disability | Age-dependent, mean 38.5 per 1000 PY (3x) | 5.83 | 19.9 | +1.1 (-0.4 to 2.6) | 0.15 |
| physical disability | No mortality (main analysis) | 2.44 | 0.0 | -6.7 (-8.6 to -4.8) | <0.001 |
| physical disability | Constant 12.8 per 1000 PY | 2.58 | 7.6 | -6.2 (-8.0 to -4.2) | <0.001 |
| physical disability | Age-dependent, mean 12.8 per 1000 PY | 2.56 | 7.3 | -6.1 (-8.0 to -4.2) | <0.001 |
| physical disability | Age-dependent, mean 25.7 per 1000 PY (2x) | 2.68 | 13.9 | -5.5 (-7.4 to -3.6) | <0.001 |
| physical disability | Age-dependent, mean 38.5 per 1000 PY (3x) | 2.81 | 19.8 | -4.9 (-6.8 to -3.0) | <0.001 |

Dates of death were not available. Person-time was reduced by applying a survival factor to each entry cohort in each calendar year. The base-case all-cause mortality (12.8 per 1,000 person-years) is that reported for Korean patients with incident seropositive RA (Choi et al., PLoS One 2019), distributed across ages by a Gompertz function doubling every eight years. Under the base case, 13% of the 2010 entrants would have died by 2022; under three times that rate, 35%.

**D. Joinpoint and quasi-Poisson analyses**

| **Analysis** | **Registered disability** | **Physical disability** |
| --- | --- | --- |
| Incidence per person-year (main analysis): best joinpoint | 2019 | 2017 |
| Annual change before / after joinpoint, % | +1.1 / −5.6 | −9.1 / −4.5 |
| p value for joinpoint (bootstrap likelihood-ratio test) | 0.11 | 0.48 |
| Proportion of prevalent RA (previous measure): best joinpoint | 2017 | 2014 |
| Annual change before / after joinpoint, % | +17.0 / +2.1 | +14.7 / +1.1 |
| p value for joinpoint | <0.001 | 0.023 |
| Quasi-Poisson annual change, % (95% CI) | −0.9 (−3.2 to 1.5) | −6.7 (−9.2 to −4.2) |
| p value; dispersion | 0.42; 2.02 | <0.001; 1.37 |
| Quasi-Poisson, men: annual change, % (p) | +0.1 (0.98) | −9.3 (<0.001) |
| Quasi-Poisson, women: annual change, % (p) | −1.5 (0.14) | −5.9 (0.001) |

Segmented Poisson regression with a single joinpoint at each candidate year between 2013 and 2020; the best-fitting model was compared with the model without a joinpoint by a parametric bootstrap likelihood-ratio test (5,000 replicates). The previous measure (annual new registrations divided by the prevalent RA population) was analyzed over 2010–2022 for comparison.
