# Supplementary material for: Nationwide trends in physical disability among rheumatoid arthritis patients in Korea (2010–2022): A retrospective cohort study based on national registry data
Source: PLoS One. 2026 Nov 4;21(11):e0362398. doi: 10.1371/journal.pone.0362398 (PMC13734594; doi:10.1371/journal.pone.0362398)
Supplement: Supplementary file 4 [file pone.0362398.s004.docx]

**S4 Table. Distribution of registered disability types among patients with seropositive rheumatoid arthritis and in the Korean general population, 2010–2022.** (A) Patients with seropositive rheumatoid arthritis. (B) General population, all ages. (C) General population aged ≥65 years.

**A. Patients with seropositive rheumatoid arthritis, n (%)**

| **Disability type** | **2010** | **2011** | **2012** | **2013** | **2014** | **2015** | **2016** | **2017** | **2018** | **2019** | **2020** | **2021** | **2022** |
| --- | --- | --- | --- | --- | --- | --- | --- | --- | --- | --- | --- | --- | --- |
| Physical | 5,903 (85.5) | 6,156 (84.9) | 6,390 (84.3) | 6,567 (83.8) | 6,837 (83.1) | 6,903 (82.3) | 7,030 (82.0) | 7,125 (80.6) | 7,188 (79.3) | 7,356 (78.0) | 7,347 (76.7) | 7,412 (75.5) | 7,406 (74.8) |
| Brain lesion | 140 (2.0) | 165 (2.3) | 179 (2.4) | 190 (2.4) | 222 (2.7) | 227 (2.7) | 228 (2.7) | 242 (2.7) | 260 (2.9) | 268 (2.8) | 270 (2.8) | 277 (2.8) | 279 (2.8) |
| Visual | 279 (4.0) | 307 (4.2) | 327 (4.3) | 357 (4.6) | 393 (4.8) | 412 (4.9) | 455 (5.3) | 486 (5.5) | 507 (5.6) | 535 (5.7) | 564 (5.9) | 600 (6.1) | 618 (6.2) |
| Hearing | 289 (4.2) | 310 (4.3) | 347 (4.6) | 381 (4.9) | 402 (4.9) | 438 (5.2) | 507 (5.9) | 612 (6.9) | 720 (7.9) | 847 (9.0) | 961 (10.0) | 1,031 (10.5) | 1,066 (10.8) |
| Speech | —ᵃ | —ᵃ | —ᵃ | 14 (0.2) | 16 (0.2) | 16 (0.2) | 17 (0.2) | 16 (0.2) | 18 (0.2) | 21 (0.2) | 25 (0.3) | 23 (0.2) | 28 (0.3) |
| Intellectual | 31 (0.4) | 41 (0.6) | 49 (0.6) | 47 (0.6) | 53 (0.6) | 57 (0.7) | 64 (0.7) | 68 (0.8) | 69 (0.8) | 74 (0.8) | 80 (0.8) | 93 (0.9) | 107 (1.1) |
| Autistic | 0 (0.0) | 0 (0.0) | 0 (0.0) | 0 (0.0) | 0 (0.0) | 0 (0.0) | 0 (0.0) | 0 (0.0) | 0 (0.0) | 0 (0.0) | 0 (0.0) | 0 (0.0) | <5 |
| Mental | 37 (0.5) | 40 (0.6) | 46 (0.6) | 51 (0.7) | 60 (0.7) | 58 (0.7) | 63 (0.7) | 64 (0.7) | 73 (0.8) | 81 (0.9) | 81 (0.8) | 81 (0.8) | 85 (0.9) |
| Kidney | 55 (0.8) | 57 (0.8) | 66 (0.9) | 70 (0.9) | 81 (1.0) | 100 (1.2) | 107 (1.2) | 126 (1.4) | 124 (1.4) | 126 (1.3) | 131 (1.4) | 162 (1.7) | 167 (1.7) |
| Cardiac | 30 (0.4) | 24 (0.3) | 20 (0.3) | 20 (0.3) | 18 (0.2) | 21 (0.3) | 17 (0.2) | 17 (0.2) | 13 (0.1) | 15 (0.2) | 14 (0.1) | 19 (0.2) | 16 (0.2) |
| Respiratory | 38 (0.6) | 38 (0.5) | 34 (0.4) | 38 (0.5) | 43 (0.5) | 46 (0.5) | 47 (0.5) | 41 (0.5) | 46 (0.5) | 53 (0.6) | 54 (0.6) | 56 (0.6) | 63 (0.6) |
| Liver | <5 | <5 | <5 | <5 | 6 (0.1) | 6 (0.1) | —ᵃ | 10 (0.1) | 12 (0.1) | 16 (0.2) | 16 (0.2) | 19 (0.2) | 18 (0.2) |
| Facial | 27 (0.4) | 31 (0.4) | 30 (0.4) | 25 (0.3) | 27 (0.3) | 27 (0.3) | <5 | <5 | <5 | <5 | <5 | <5 | 0 (0.0) |
| Ostomy | 48 (0.7) | 54 (0.7) | 58 (0.8) | 63 (0.8) | 61 (0.7) | 66 (0.8) | 21 (0.2) | 23 (0.3) | 28 (0.3) | 25 (0.3) | 30 (0.3) | 32 (0.3) | 34 (0.3) |
| Epilepsy | 12 (0.2) | 17 (0.2) | 16 (0.2) | —ᵃ | 12 (0.1) | 13 (0.2) | 8 (0.1) | —ᵃ | —ᵃ | —ᵃ | —ᵃ | —ᵃ | —ᵃ |
| Total, n | 6,901 | 7,253 | 7,576 | 7,841 | 8,231 | 8,390 | 8,573 | 8,838 | 9,069 | 9,427 | 9,583 | 9,815 | 9,899 |

Main disability type recorded in the registry among patients with a registered disability in the prevalent RA population. Counts of fewer than 5 patients are shown as <5. ᵃSuppressed so that a count of fewer than 5 cannot be derived from the total.

**B. General population, all ages, %**

| **Disability type** | **2010** | **2011** | **2012** | **2013** | **2014** | **2015** | **2016** | **2017** | **2018** | **2019** | **2020** | **2021** | **2022** |
| --- | --- | --- | --- | --- | --- | --- | --- | --- | --- | --- | --- | --- | --- |
| Physical | 53.1 | 52.9 | 52.7 | 52.3 | 51.9 | 51.5 | 50.5 | 49.3 | 47.9 | 46.7 | 45.9 | 45.1 | 44.4 |
| Brain lesion | 10.4 | 10.3 | 10.3 | 10.1 | 10.1 | 10.1 | 10.0 | 9.9 | 9.8 | 9.6 | 9.5 | 9.4 | 9.2 |
| Visual | 9.9 | 10.0 | 10.1 | 10.1 | 10.1 | 10.2 | 10.1 | 9.9 | 9.8 | 9.7 | 9.6 | 9.5 | 9.5 |
| Hearing | 10.3 | 10.4 | 10.3 | 10.2 | 10.1 | 10.1 | 10.8 | 11.9 | 13.2 | 14.4 | 15.0 | 15.6 | 16.0 |
| Speech | 0.7 | 0.7 | 0.7 | 0.7 | 0.7 | 0.8 | 0.8 | 0.8 | 0.8 | 0.8 | 0.9 | 0.9 | 0.9 |
| Intellectual | 6.4 | 6.6 | 6.9 | 7.2 | 7.4 | 7.6 | 7.8 | 7.9 | 8.0 | 8.1 | 8.2 | 8.4 | 8.5 |
| Autistic | 0.6 | 0.6 | 0.7 | 0.7 | 0.8 | 0.8 | 0.9 | 1.0 | 1.0 | 1.1 | 1.2 | 1.3 | 1.4 |
| Mental | 3.8 | 3.8 | 3.8 | 3.8 | 3.9 | 4.0 | 4.0 | 4.0 | 3.9 | 3.9 | 3.9 | 3.9 | 3.9 |
| Kidney | 2.3 | 2.4 | 2.5 | 2.7 | 2.8 | 3.0 | 3.1 | 3.3 | 3.4 | 3.5 | 3.7 | 3.9 | 4.0 |
| Cardiac | 0.5 | 0.4 | 0.3 | 0.3 | 0.3 | 0.2 | 0.2 | 0.2 | 0.2 | 0.2 | 0.2 | 0.2 | 0.2 |
| Respiratory | 0.6 | 0.6 | 0.6 | 0.5 | 0.5 | 0.5 | 0.5 | 0.5 | 0.5 | 0.4 | 0.4 | 0.4 | 0.4 |
| Liver | 0.3 | 0.3 | 0.3 | 0.4 | 0.4 | 0.4 | 0.4 | 0.5 | 0.5 | 0.5 | 0.5 | 0.5 | 0.6 |
| Facial | 0.1 | 0.1 | 0.1 | 0.1 | 0.1 | 0.1 | 0.1 | 0.1 | 0.1 | 0.1 | 0.1 | 0.1 | 0.1 |
| Ostomy | 0.5 | 0.5 | 0.5 | 0.5 | 0.6 | 0.6 | 0.6 | 0.6 | 0.6 | 0.6 | 0.6 | 0.6 | 0.6 |
| Epilepsy | 0.4 | 0.4 | 0.3 | 0.3 | 0.3 | 0.3 | 0.3 | 0.3 | 0.3 | 0.3 | 0.3 | 0.3 | 0.3 |
| Total, n | 2,517,312 | 2,519,241 | 2,511,159 | 2,501,111 | 2,494,459 | 2,490,406 | 2,511,050 | 2,545,637 | 2,585,876 | 2,618,918 | 2,633,026 | 2,642,922 | 2,648,424 |

Calculated from the annual disability registration statistics of the Ministry of Health and Welfare.

**C. General population aged ≥65 years, %**

| **Disability type** | **2010** | **2011** | **2012** | **2013** | **2014** | **2015** | **2016** | **2017** | **2018** | **2019** | **2020** | **2021** | **2022** |
| --- | --- | --- | --- | --- | --- | --- | --- | --- | --- | --- | --- | --- | --- |
| Physical | 52.0 | 52.4 | 52.8 | 53.3 | 53.6 | 53.7 | 52.8 | 51.4 | 49.7 | 48.5 | 47.9 | 47.5 | 47.1 |
| Brain lesion | 14.5 | 14.2 | 13.8 | 13.4 | 13.1 | 12.9 | 12.5 | 12.1 | 11.6 | 11.1 | 10.8 | 10.4 | 10.1 |
| Visual | 10.8 | 11.0 | 11.1 | 11.2 | 11.3 | 11.3 | 11.1 | 10.8 | 10.5 | 10.2 | 10.1 | 9.9 | 9.8 |
| Hearing | 16.5 | 16.4 | 16.1 | 15.8 | 15.6 | 15.4 | 16.8 | 18.5 | 20.9 | 22.7 | 23.5 | 24.1 | 24.5 |
| Speech | 0.6 | 0.6 | 0.6 | 0.6 | 0.6 | 0.6 | 0.6 | 0.6 | 0.6 | 0.6 | 0.6 | 0.6 | 0.6 |
| Intellectual | 0.6 | 0.6 | 0.6 | 0.7 | 0.7 | 0.7 | 0.8 | 0.8 | 0.8 | 0.8 | 0.9 | 0.9 | 1.0 |
| Autistic | 0.0 | 0.0 | 0.0 | 0.0 | 0.0 | 0.0 | 0.0 | 0.0 | 0.0 | 0.0 | 0.0 | 0.0 | 0.0 |
| Mental | 0.8 | 0.8 | 0.8 | 0.8 | 0.9 | 0.9 | 1.0 | 1.1 | 1.1 | 1.2 | 1.3 | 1.4 | 1.5 |
| Kidney | 1.7 | 1.8 | 1.9 | 2.0 | 2.2 | 2.3 | 2.4 | 2.6 | 2.7 | 2.8 | 3.0 | 3.1 | 3.3 |
| Cardiac | 0.6 | 0.5 | 0.4 | 0.3 | 0.3 | 0.3 | 0.2 | 0.2 | 0.2 | 0.2 | 0.2 | 0.2 | 0.2 |
| Respiratory | 0.9 | 0.8 | 0.8 | 0.7 | 0.7 | 0.7 | 0.6 | 0.6 | 0.6 | 0.6 | 0.6 | 0.6 | 0.6 |
| Liver | 0.1 | 0.1 | 0.1 | 0.1 | 0.2 | 0.2 | 0.2 | 0.2 | 0.3 | 0.3 | 0.3 | 0.4 | 0.4 |
| Facial | 0.0 | 0.0 | 0.0 | 0.0 | 0.0 | 0.0 | 0.0 | 0.1 | 0.1 | 0.1 | 0.1 | 0.1 | 0.1 |
| Ostomy | 0.8 | 0.8 | 0.8 | 0.9 | 0.9 | 0.9 | 0.9 | 0.9 | 0.9 | 0.8 | 0.8 | 0.8 | 0.8 |
| Epilepsy | 0.1 | 0.1 | 0.0 | 0.0 | 0.0 | 0.1 | 0.1 | 0.1 | 0.1 | 0.1 | 0.1 | 0.1 | 0.1 |
| Total, n | 934,634 | 957,824 | 987,251 | 1,009,871 | 1,033,307 | 1,053,364 | 1,089,316 | 1,149,910 | 1,206,482 | 1,263,952 | 1,314,625 | 1,355,712 | 1,397,669 |

Calculated from the annual disability registration statistics of the Ministry of Health and Welfare.
